# The essential roles of lncRNAs/PI3K/AKT axis in gastrointestinal tumors

**DOI:** 10.3389/fcell.2024.1442193

**Published:** 2024-08-05

**Authors:** Penghui Li, Xiao Ma, Xinyu Gu

**Affiliations:** ^1^ Department of Gastrointestinal Surgery, The First Affiliated Hospital, College of Clinical Medicine, Henan University of Science and Technology, Luoyang, China; ^2^ Zhejiang University School of Medicine, Hangzhou, China; ^3^ Department of Oncology, The First Affiliated Hospital, College of Clinical Medicine, Henan University of Science and Technology, Luoyang, China

**Keywords:** lncRNAs, PI3K/Akt pathway, gastrointestinal tumors, expression features, mechanisms

## Abstract

The role of long noncoding RNA (lncRNA) in tumors, particularly in gastrointestinal tumors, has gained significant attention. Accumulating evidence underscores the interaction between various lncRNAs and diverse molecular pathways involved in cancer progression. One such pivotal pathway is the PI3K/AKT pathway, which serves as a crucial intracellular mechanism maintaining the balance among various cellular physiological processes for normal cell growth and survival. Frequent dysregulation of the PI3K/AKT pathway in cancer, along with aberrant activation, plays a critical role in driving tumorigenesis. LncRNAs modulate the PI3K/AKT signaling pathway through diverse mechanisms, primarily by acting as competing endogenous RNA to regulate miRNA expression and associated genes. This interaction significantly influences fundamental biological behaviors such as cell proliferation, metastasis, and drug resistance. Abnormal expression of numerous lncRNAs in gastrointestinal tumors often correlates with clinical outcomes and pathological features in patients with cancer. Additionally, these lncRNAs influence the sensitivity of tumor cells to chemotherapy in multiple types of gastrointestinal tumors through the abnormal activation of the PI3K/AKT pathway. These findings provide valuable insights into the mechanisms underlying gastrointestinal tumors and potential therapeutic targets. However, gastrointestinal tumors remain a significant global health concern, with increasing incidence and mortality rates of gastrointestinal tumors over recent decades. This review provides a comprehensive summary of the latest research on the interactions of lncRNA and the PI3K/AKT pathway in gastrointestinal tumor development. Additionally, it focuses on the functions of lncRNAs and the PI3K/AKT pathway in carcinogenesis, exploring expression profiles, clinicopathological characteristics, interaction mechanisms with the PI3K/AKT pathway, and potential clinical applications.

## 1 Introduction

Long noncoding RNAs (lncRNAs) are a set of noncoding RNAs comprising more than 200 nucleotides in length and constitute approximately 60% of human cell transcriptional output (Mattick and Makunin, 2006; Djebali et al., 2012; Tao et al., 2023). As RNA sequencing methods and epigenomic techniques advance, novel lncRNA types and their distinct cellular functions continue to emerge (Anastasiadou et al., 2018b; Qian et al., 2019; Saw et al., 2021; Herman et al., 2022). Despite lacking protein-coding ability, lncRNAs play crucial roles in regulating gene expression, maintaining chromosomal stability, and influencing alternative splicing, depending on their cellular location (Bridges et al., 2021; Tan et al., 2021; Zhao et al., 2022). Recent studies highlight their significance as regulators of diverse physiological and pathological processes (Romeo et al., 2023). Interacting with various RNAs, lncRNAs affect multiple molecular targets, modulating their stability and downstream gene expression (Kopp and Mendell, 2018; Shaath et al., 2022; Yao et al., 2022; Li et al., 2024). Aberrant lncRNA expressions have been identified across various human tumors and diseases, spanning gastric cancer (GC), breast cancer, liver cancer (LC), melanoma, and cardiovascular diseases (Anastasiadou et al., 2018a; Yan and Bu, 2021). Given the distinct expression profiles and association with patient prognosis, various lncRNAs serve as potent biomarkers for diagnosing and predicting human disorders (Ozawa et al., 2017; Peng et al., 2018; Costa et al., 2023). In particular, lncRNAs have emerged as critical regulators and exert their functions mainly through intricate competing endogenous RNA (ceRNA) networks in gastrointestinal tumors including GC, LC, colorectal cancer (CRC), pancreatic cancer (PC), and esophageal cancer (EC) (Guo et al., 2015; Chan and Tay, 2018; Wang et al., 2019a; Mo et al., 2022; Xue et al., 2023). The expression levels of certain lncRNAs, whether upregulated or downregulated, are strongly associated with the clinicopathological characteristics of patients with gastrointestinal tumors (Xiao et al., 2021; Lulli et al., 2022; Wei et al., 2022). Dysregulated lncRNAs actively contribute to cell proliferation, migration, invasion, epithelial-to-mesenchymal transition (EMT), apoptosis, and drug resistance during the initiation and development of multiple gastrointestinal tumors (Panzitt et al., 2007; Wang X. et al., 2017; Zheng et al., 2018; Wang N. et al., 2019; Lv et al., 2021). As RNA-based therapeutics gain traction in clinical settings, understanding intricate lncRNA mechanisms is essential for the effective diagnosis and treatment of gastrointestinal tumors (Ozawa et al., 2017; Wang et al., 2019b; Sun et al., 2023).

**FIGURE 1:**
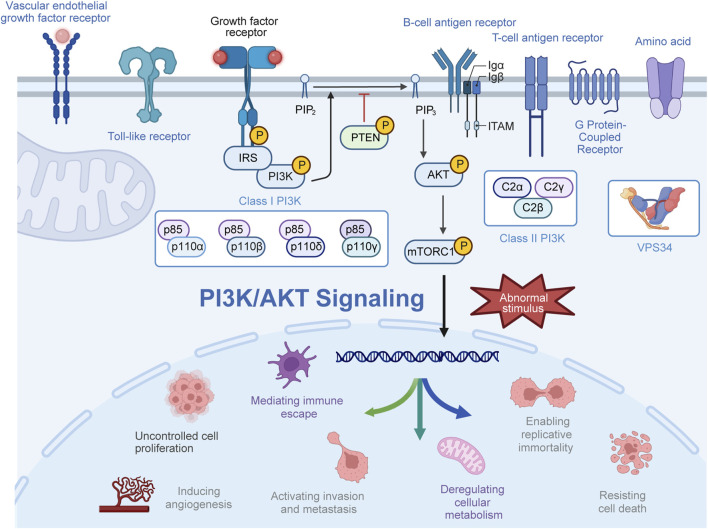
Activation process of the PI3K/AKT pathway. Upon activation by receptor-coupled tyrosine kinases in response to extracellular stimuli, PI3K phosphorylates phosphatidylinositol 4,5-bisphosphate (PIP2) to generate phosphatidylinositol 3,4,5-trisphosphate (PIP3). Subsequently, PI3P recruits and phosphorylates AKT at the plasma membrane, thereby exerting a spectrum of biological effects.

The phosphatidylinositol 3-kinase (PI3K) pathway serves as a crucial intracellular signaling pathway governing various cellular processes across different tissues, including cell growth, proliferation, metabolism, migration, and immune response (Katso et al., 2001; Engelman et al., 2006; Xue et al., 2021). PI3K, a pivotal lipid kinase, can be activated through multiple upstream pathways, giving rise to downstream effects (MacDougall et al., 1995; Vanhaesebroeck et al., 1997). In humans, three classes of PI3Ks—class I, II, and III—exist, with an extensive focus on class I PI3Ks (Vanhaesebroeck et al., 2010; Okkenhaug, 2013; Jean and Kiger, 2014). Class I PI3Ks form heterodimers from the merging of p110 catalytic subunits and p85 regulatory subunits (Cui et al., 2014; Zhang et al., 2020; Hart et al., 2022). Four isoforms of p110—p110α, p110β, p110γ, and p110δ—encoded by PIK3CA, PIK3CB, PIK3CG, and PIK3CD, respectively, are part of this class. The regulatory subunit p85 exists in three forms, namely, p85α, p85β, and p85γ, with p85α being the most abundantly expressed regulatory subunit (Haug et al., 1991; Fox et al., 2020; Chaudhuri et al., 2023). Class II PI3Ks lack regulatory subunits, primarily characterized as monomer enzymes. Class II PI3Ks exist as three catalytic subtypes, including PI3KC2α and PI3KC2β, which are widely distributed, while PI3KC2γ is specifically found in the liver (Gulluni et al., 2019; Thibault et al., 2023). Moreover, VPS34, the sole member of class III PI3K, forms tetrameric complexes I and II with regulatory and catalytic subunits (Backer, 2008; Reidick et al., 2017; Ohashi et al., 2019; Alkhoury et al., 2023). Generally, mutations in the PI3K gene, amplifications, increased activity of oncogenic proteins upstream, and loss of tumor suppressor genes all contribute to elevated PI3K activity in tumors. Activation of PI3K occurs through the combination of receptor tyrosine kinases and various growth factor ligands such as platelet-derived growth factor, epidermal growth factor (EGF), and insulin-like growth factor (IGF) (Chang et al., 2003). Activated PI3K generates phosphatidylinositol 3,4,5-trisphosphate (PIP3) from phosphatidylinositol 4,5-bisphosphate (PIP2), recruiting downstream signaling proteins to activate downstream signaling pathways such as AGC serine/threonine kinases (AKT) (Calamito et al., 2010; Chin and Toker, 2011). Subsequently, AKT phosphorylates downstream effectors in a sequence-dependent manner, influencing a wide array of functions, commonly recognizing the motif (R-X-R-X-X-S/T; Figure 1) (Obata et al., 2000; Nag et al., 2023; Zhou et al., 2023).

Hyperactivation of the PI3K pathway has been extensively studied in multiple tumors, highlighting its integral role in tumor development and progression (Altomare and Testa, 2005; Ediriweera et al., 2019; Miricescu et al., 2020). In addition, PI3K mutations, particularly in the PIK3CA gene, are frequently observed in colorectal, gastric, and liver cancers. These mutations lead to constitutive activation of the PI3K-AKT pathway, promoting uncontrolled cell proliferation, survival, metastasis, and invasion (Huang D. et al., 2018; Liu W. et al., 2022; Chong et al., 2022). Animal models have been instrumental in elucidating the role of PI3K-AKT pathway mutations in gastrointestinal tumors. For instance, transgenic mice with hepatocyte-specific PTEN deficiency leads to the development of liver tumors, highlights the oncogenic potential of PI3K activation (Horie et al., 2004). Similarly, mouse models with intestinal epithelial-specific PIK3CA mutations exhibit increased tumorigenesis, supporting the role of PI3K-Akt signaling in colorectal cancer development (Cathomas, 2014; Chen et al., 2022; Wang et al., 2023). Therapeutic strategies targeting the PI3K pathway, owing to the core role of PI3K signaling in numerous tumor pathogenic processes, are rapidly evolving and entering preclinical and early clinical trials (Beck et al., 2014; Alzahrani, 2019; Tewari et al., 2022; Yu et al., 2022). Dysregulation of the PI3K pathway, stimulated by various factors such as epigenetic alterations and environmental influences, is observed in gastrointestinal tumors, contributing to uncontrolled cell proliferation, survival, cell cycle progression, resistance to apoptosis, and chemoresistance (Chang et al., 2003; Danielsen et al., 2015; Papadatos-Pastos et al., 2015; Liu R. et al., 2020). The PI3K pathway’s involvement in mediating the oncogenic effects of several lncRNAs has been identified to contribute to tumor growth and metastasis in gastrointestinal tumors (Wang Y. et al., 2020; Zhao et al., 2020; Fang L. et al., 2022). Preclinical and clinical studies have highlighted the potential of targeting the PI3K pathway as a therapeutic intervention for gastrointestinal tumors mainly based on the influence on tumor resistance to antineoplastic drugs (Ng et al., 2000; O’gorman and Cotter, 2001; Mueller et al., 2012; Du J. et al., 2022). Continued exploration of the mechanisms underlying PI3K pathway dysregulation will aid in developing targeted therapies, potentially improving outcomes for patients with gastrointestinal tumors (Michl and Downward, 2005; Zhang et al., 2011; Su et al., 2022).

This review comprehensively outlines the dysregulated expression patterns of lncRNAs and their correlation with clinicopathological features in patients with gastrointestinal tumors. Additionally, we also elucidate the mechanisms underlying the crosstalk between lncRNAs and the PI3K/AKT pathway in gastrointestinal tumors. Given the pivotal role of this interaction, the potential implications for precise diagnosis, prognosis, and therapeutic interventions have been explored.

## 2 Involvement of lncRNAs and PI3/AKT pathway in gastrointestinal tumors

**FIGURE 2:**
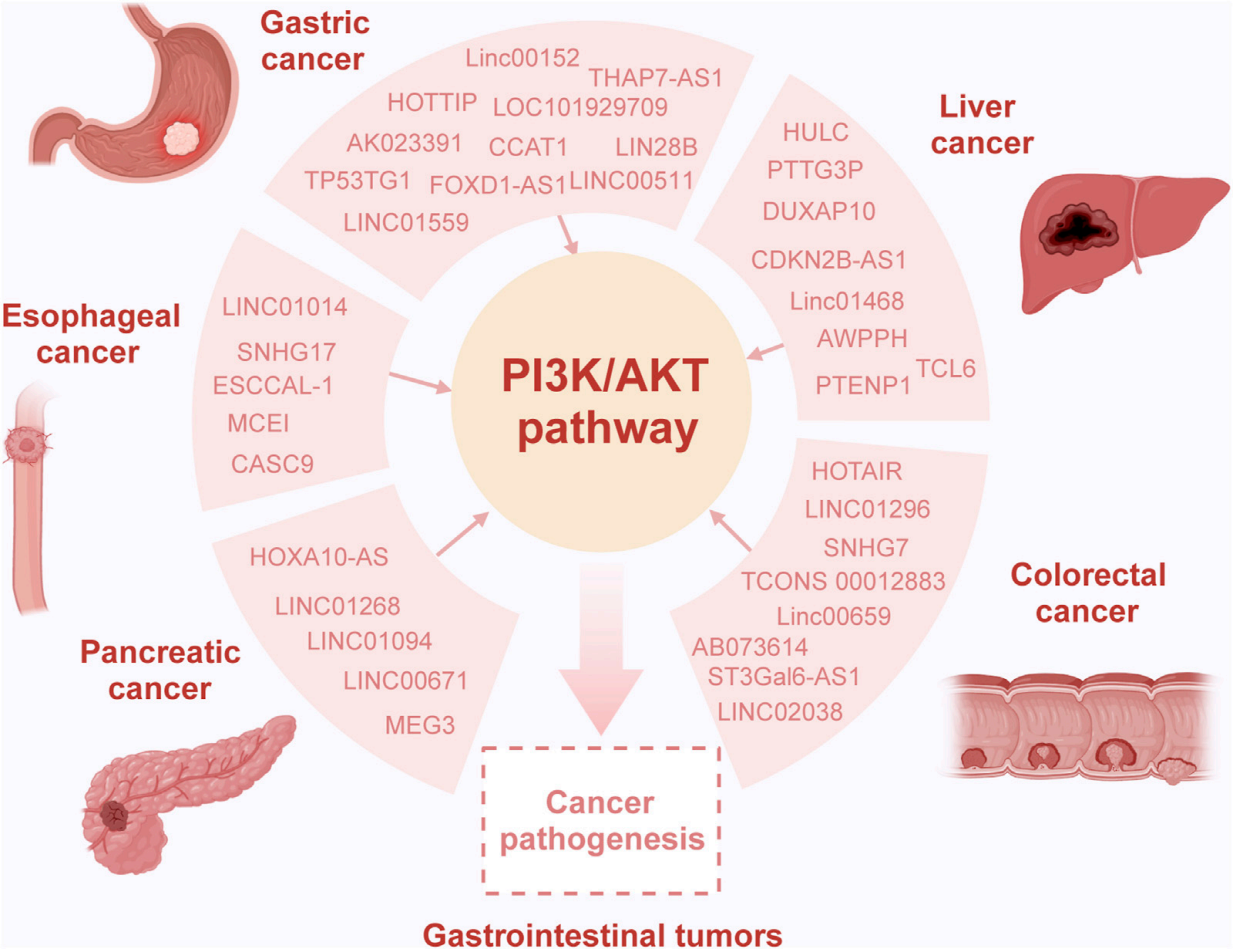
Overview of the interaction of lncRNA and the PI3K/AKT pathway in gastrointestinal tumors. Dysregulation of long non-coding RNAs (lncRNAs) is common in gastrointestinal tumors, playing intricate roles in both oncogenic and tumor-suppressor processes contingent upon the specific molecular interactions in the context of cancer. Specifically, various lncRNAs act as sponges, binding completely with miRNAs, consequently modulating target gene expression and influencing the activity of the PI3K/AKT pathway. This altered pathway activity is considered pivotal in mediating the oncogenic effects induced by lncRNAs, contributing to aberrant cellular processes and tumor progression.

Recent research indicates that lncRNAs can modulate the activity of the PI3K signaling pathway through various mechanisms, thereby influencing the development of gastrointestinal tumors (Table 1) (Shang et al., 2019; Luo J. et al., 2021; Almalki, 2023). Numerous studies have highlighted the role of lncRNAs as competing endogenous RNAs (ceRNAs), where they competitively bind to microRNAs (miRNAs), mediating the expression of downstream target genes and regulating the PI3K signaling pathway (Table 2) (Wei et al., 2019; Rahmani et al., 2020; Kumar et al., 2023). Additionally, the PI3K signaling pathway can influence the expression levels of lncRNAs through a series of downstream effector proteins (Dai et al., 2020; Zhong et al., 2022). The abnormal interaction between lncRNAs and the PI3K/AKT pathway ultimately affects multiple cellular processes and tumor progression. This section emphasizes the molecular mechanisms by which lncRNAs modulate the oncogenic PI3K/AKT pathway, impacting various biological processes in gastrointestinal tumors, including cell proliferation, migration, invasion, and chemoresistance (Figure 2) (He et al., 2021).

**TABLE 1:** Roles of lncRNA/PI3K/AKT axis in gastrointestinal tumors.

Cancer type	LncRNA	Roles	Related clinical features	Clinical value	Refs
Gastric cancer	LncRNA AK023391	oncogenic properties	poor survival	an independent prognostic factor	Huang et al. (2017)
Gastric cancer	LncRNA THAP7-AS1	oncogenic properties	positive lymph node metastasis, higher ACJJ stage, and poor survival	distinguish GC cases with and without lymph node metastasis	Liu et al. (2022a)
Gastric cancer	Linc00152	oncogenic properties	bigger tumor size	—	Zhou et al. (2015)
Gastric cancer	LINC01559	oncogenic properties	—	—	Wang et al. (2020a)
Gastric cancer	LOC101929709, and LIN28B	oncogenic properties	—	—	Xu et al. (2023)
Gastric cancer	LncRNA HOTTIP	oncogenic molecule	—	—	Xin et al. (2023)
Gastric cancer	LncRNA CCAT1	oncogenic molecule	advanced tumor diameter, venous/lymphatic invasion, and biochemical indexes	—	Song et al. (2020)
Gastric cancer	LncRNA FOXD1-AS1	oncogenic molecule	—	—	Wu et al. (2021a)
Gastric cancer	LINC00511	oncogenic molecule	—	—	Wang et al. (2021)
Gastric cancer	LncRNA TP53TG1	tumor suppressor	improved prognosis, tumor diameter, differentiation, TNM categories, and lymph node metastasis	a potential index for the early diagnosis and prognosis	Fang et al. (2022a)
Liver cancer	LncRNA HULC	oncogenic molecule	—	a novel therapeutic approach	Xin et al. (2018)
Liver cancer	LncRNA HULC	oncogenic molecule	—	—	Wang et al. (2020b)
Liver cancer	LncRNA PTTG3P	oncogenic molecule	—	an independent prognostic factor	Huang et al. (2018b)
Liver cancer	LncRNA DUXAP10	oncogenic molecule	—	—	Sun et al. (2019)
Liver cancer	LncRNA CDKN2B-AS1	oncogenic molecule	large tumor size, microvascular invasion, high tumor grade, advanced tumor stage, and reduced survival	—	Huang et al. (2018d)
Liver cancer	Linc01468	oncogenic molecule	unfavorable hemoglobin A1C, triglyceride, and total cholesterol, and cirrhosis levels, tumor size, tumor stage, TNM stage, microvascular invasion, and lower overall survival	—	Wang et al. (2022a)
Liver cancer	LncRNA AWPPH	oncogenic molecule	encapsulation incomplete, microvascular invasion, advanced TNM stage and BCLC stage, and poor recurrence-free and overall survival	an independent prognostic factor	Zhao et al. (2017)
Liver cancer	LncRNA TCL6	tumor suppressor	—	—	Luo et al. (2020)
Liver cancer	LncRNA PTENP1	tumor suppressor	—	a novel therapeutic approach	Chen et al. (2015)
Hepatoblastoma	LncRNA MIR205HG	oncogenic molecule	—	—	Zhang et al. (2022)
Cholangiocarcinoma	LncRNA HCG18	oncogenic molecule		—	Ni et al. (2023)
Colorectal cancer	LINC01296	oncogenic molecule	advanced tumor stage, distant metastasis, and poor prognosis	—	Liu et al. (2018)
Colorectal cancer	LncRNA HOTAIR	oncogenic molecule	poor prognosis	—	Pan et al. (2019)
Colorectal cancer	LncRNA TCONS_00012883	oncogenic molecule	larger tumor size, tumor stages, lymph node metastasis, shorter overall survival, and progression-free survival	—	Yang et al. (2020)
Colorectal cancer	Linc00659	oncogenic molecule	poor prognosis	—	Tsai et al. (2018)
Colorectal cancer	LncRNA SNHG7	oncogenic molecule	poor overall survival and progression-free survival, advanced tumor size, lymphatic metastasis, distant metastasis, and tumor stage	—	Li et al. (2018)
Colorectal cancer	LncRNA MALAT1	oncogenic molecule	—	—	Xu et al. (2020)
Colorectal cancer	LncRNA AB073614	oncogenic molecule	advanced tumor grade, size, cell differentiation status, and the presence of distant metastases	—	Wang et al. (2017b)
Colorectal cancer	LncRNA ST3Gal6-AS1	tumor suppressor	favorable tumor size, lymphatic metastasis, distant metastasis, and tumor stage	—	Hu et al. (2019)
Colorectal cancer	LINC02038	tumor suppressor	favorable TNM stage, lymphatic metastasis and distant metastasis, and overall survival	—	Liu et al. (2023c)
Pancreatic cancer	LINC01268	oncogenic molecule	—	—	Liu et al. (2023b)
Pancreatic cancer	LncRNA HOXA10-AS	oncogenic molecule	poor prognosis	—	Wu et al. (2022)
Pancreatic cancer	LINC01094	oncogenic molecule	poor prognosis, unfavorable tumor size, lymphatic metastasis, andTNM classification	an independent prognostic factor	Luo et al. (2021a)
Pancreatic cancer	LncRNA MEG3	tumor suppressor	favorable tumor size, metastasis, and vascular invasion	—	Gu et al. (2017)
Pancreatic cancer	LINC00671	tumor suppressor	favorable tumor differentiation, aggressiveness, and prognosis	—	Qu et al. (2021)
Esophageal cancer	LINC01014	oncogenic molecule	—	—	Fu et al. (2020)
Esophageal cancer	LncRNA MCEI	oncogenic molecule	poor survival	—	Liu et al. (2020a)
Esophageal cancer	LncRNA CASC9	oncogenic molecule	advanced tumor differentiation grade, primary tumor invasion depth, lymph node metastasis, and TNM stage	—	Liang et al. (2018)
Esophageal cancer	LncRNA SNHG17	oncogenic molecule	poor survival, advanced TNM stage, depth of invasion, tumor differentiation, lymph node metastasis, and mortality	an independent prognostic factor	Shen et al. (2022)
Esophageal cancer	LncRNA ESCCAL-1	oncogenic molecule	poor survival, TNM stage, and lymph node metastasis	—	Liu et al. (2020b)

**TABLE 2:** Molecular mechanisms of lncRNA and PI3K/AKT pathway in gastrointestinal tumors.

Cancer type	LncRNA	Related mechanisms	Expression	Function	Refs
Gastric cancer	LncRNA AK023391	NF-κB, FOXO3a, c-myb, cyclinB1/g2, bcl-6, and p53	upregulated	promote cell growth and invasion, inhibit apoptosis and cell cycle arrest	Huang et al. (2017)
Gastric cancer	LncRNA THAP7-AS1	SP1, CUL4B, miR-22-3p, and miR-320a	upregulated	promote cell growth, invasion and metastasis	Liu et al. (2022a)
Gastric cancer	Linc00152	EGFR	upregulated	promote cell proliferation	Zhou et al. (2015)
Gastric cancer	LINC01559	miR-1343-3p, PGK1, and PTEN	upregulated	promote cell proliferation, migration, and stemness	Wang et al. (2020a)
Gastric cancer	LOC101929709, and LIN28B	c-MYC	upregulated	promote cell proliferation, migration, and glycolysis	Xu et al. (2023)
Gastric cancer	LncRNA HOTTIP	miRNA-885-3p, and EphB2	upregulated	promote cell proliferation, migration, invasion, tumor growth, and metastasis	Xin et al. (2023)
Gastric cancer	LncRNA CCAT1	CTD	upregulated	promote cell growth, migration, and invasion, inhibit cell apoptosis	Song et al. (2020)
Gastric cancer	LncRNA FOXD1-AS1	FOXD1, miR-466, and eIF4G-eIF4E-eIF4A translational complex	upregulated	promote cell proliferation, motility, cisplatin resistance, tumor growth, and metastasis	Wu et al. (2021a)
Gastric cancer	LINC00511	SOX4, miR-195-5p, EZH2, and PTEN	upregulated	promote cell migration, EMT, stemness, and tumor growth	Wang et al. (2021)
Gastric cancer	LncRNA TP53TG1	ALKBH5, and CIP2A	downregulated	inhibit cell proliferation, metastasis, and cell cycle progression, promote cell apoptosis	Fang et al. (2022a)
Liver cancer	LncRNA HULC	miR15a, Sirt1, P62, and PTEN	upregulated	promote cell autophagy, and proliferation	Xin et al. (2018)
Liver cancer	LncRNA HULC	miR24-2	upregulated	promote cell autophagy, and growth	Wang et al. (2020b)
Liver cancer	LncRNA PTTG3P	PTTG1	upregulated	promote cell proliferation, migration, invasion, tumorigenesis and metastasis	Huang et al. (2018b)
Liver cancer	LncRNA DUXAP10	miR-1914, and GPR39	upregulated	promote cell proliferation, cell cycle progression, and colony formation, inhibit apoptosis	Sun et al. (2019)
Liver cancer	LncRNA CDKN2B-AS1	let-7c-5p, and NAP1L1	upregulated	promote cell proliferation, migration, and invasion, cell cycle progression, tumor growth, and metastasis, inhibit apoptosis	Huang et al. (2018d)
Liver cancer	Linc01468	SHIP2, and CUL4A	upregulated	promote lipogenesis, cell proliferation, and sorafenib resistance	Wang et al. (2022a)
Liver cancer	LncRNA AWPPH	YBX1	upregulated	promote cell proliferation and migration, tumor growth and metastasis	Zhao et al. (2017)
Liver cancer	LncRNA TCL6	miR-106a-5p, and PTEN	downregulated	inhibit cell proliferation, migration, and invasion	Luo et al. (2020)
Liver cancer	LncRNA PTENP1	PTEN	downregulated	inhibit tumor growth, angiogenesis, cell proliferation, migration, invasion, and promote cell apoptosis, and autophagy	Chen et al. (2015)
Hepatoblastoma	LncRNA MIR205HG	miR-514a-5p	upregulated	promote cell proliferation, migration, and invasion	Zhang et al. (2022)
Cholangiocarcinoma	LncRNA HCG18	miR-424-5p, and SOX9	upregulated	promote cell proliferation, migration, and invasion	Ni et al. (2023)
Colorectal cancer	LINC01296	miR-26a, GALNT3, and MUC1	upregulated	promote tumorigenesis, liver metastasis, and cell chemoresistance	Liu et al. (2018)
Colorectal cancer	LncRNA HOTAIR	miR-326, FUT6, and CD44	upregulated	promote cell proliferation, migration, invasion, tumorigenesis, and liver metastasis	Pan et al. (2019)
Colorectal cancer	LncRNA TCONS_00012883	DDX3, YY1, and MMP1	upregulated	promote cell proliferation and metastasis, tumor growth and metastasis	Yang et al. (2020)
Colorectal cancer	Linc00659	—	upregulated	promote cell cycle progression, cell proliferation, drug resistance, and colony formation	Tsai et al. (2018)
Colorectal cancer	LncRNA SNHG7	miR-34a, and GALNT7	upregulated	promote cell proliferation, metastasis, mediate cell cycle, and inhibit apoptosis	Li et al. (2018)
Colorectal cancer	LncRNA MALAT1	miR-26a/26b, and FUT4	upregulated	promote cell proliferation, metastasis, and invasion	Xu et al. (2020)
Colorectal cancer	LncRNA AB073614	—	upregulated	promote cell proliferation, migration, invasion, and cell cycle progression, and inhibit apoptosis	Wang et al. (2017b)
Colorectal cancer	LncRNA ST3Gal6-AS1	ST3Gal6, and Foxo1	downregulated	inhibit cell proliferation, metastasis, and promote cell apoptosis	Hu et al. (2019)
Colorectal cancer	LINC02038	miR-552-5p, and FAM172A	downregulated	inhibit cell proliferation, vitality, migration, and invasion	Liu et al. (2023c)
Pancreatic cancer	LINC01268	miR-217-KIF2A	upregulated	promote cell proliferation, migration, invasion, and EMT	Liu et al. (2023b)
Pancreatic cancer	LncRNA HOXA10-AS	miR-340-3p, and HTR1D	upregulated	promote cell proliferation, migration, and invasion, and inhibit apoptosis	Wu et al. (2022)
Pancreatic cancer	LINC01094	miR-577, and LIN28B	upregulated	promote cell proliferation, migration, and invasion, and inhibit apoptosis	Luo et al. (2021a)
Pancreatic cancer	LncRNA MEG3	—	downregulated	inhibit cell proliferation, migration, and invasion	Gu et al. (2017)
Pancreatic cancer	LINC00671	—	downregulated	suppress cell proliferation, metastasis, and tumor growth	Qu et al. (2021)
Esophageal cancer	LINC01014	—	upregulated	promote gefitinib resistance	Fu et al. (2020)
Esophageal cancer	LncRNA MCEI	miR-6759-5p, and IGF2	upregulated	promote cisplatin resistance, cell proliferation, colony formation, migration, and invasion, and inhibit apoptosis	Liu et al. (2020a)
Esophageal cancer	LncRNA CASC9	LAMC2, and FAK	upregulated	promote cell migration, and invasion	Liang et al. (2018)
Esophageal cancer	LncRNA SNHG17	TGF-β1, and c-Myc	upregulated	promote cell proliferation, migration, EMT, and invasion, and inhibit apoptosis	Shen et al. (2022)
Esophageal cancer	LncRNA ESCCAL-1	APOBEC3G	upregulated	promote cell proliferation, migration, and invasion, and inhibit apoptosis	Liu et al. (2020b)

### 2.1 Gastric cancer

In GC, upregulated expression of the lncRNA AK023391 was observed in clinical samples and cell lines, and the expression level was positively associated with poor overall survival (OS) among patients with GC. Both *in vitro* and *in vivo* experiments demonstrated that overexpression of AK023391 prompted cell growth, colony formation, and invasion and hindered cell apoptosis and cell cycle arrest. Mechanistic exploration revealed that the lncRNA AK023391 interacted with the PI3K/AKT signaling pathway, subsequently triggering NF-κB and inactivating FOXO3a to exert its oncogenic effects. This was accompanied by upregulated expression of c-myb, cyclinB1/g2, and bcl-6, as well as downregulation of the p53 pathways in GC (Huang et al., 2017). Furthermore, the lncRNA THAP7-AS1 exhibited overexpression in GC tissues with lymph node metastasis, and it is closely associated with the poorer American Joint Committee on Cancer staging and prognosis. Analysis using receiver operating characteristic curves indicated that the lncRNA THAP7-AS1 effectively distinguished between GC cases with and without lymph node metastasis (area under the curve = 0.7367). Cellular experiments demonstrated that high expression of THAP7-AS1 promoted the proliferation, invasion, and migration abilities of GC cells (Liu J. et al., 2023). Mechanistic investigations unveiled that the transcription factor SP1 activates the transcription of THAP7-AS1, while METTL3 mediates m6A modification of THAP7-AS1 through an IGF2BP1-dependent mechanism, enhancing its expression level. Upregulated expression of THAP7-AS1 facilitates the nuclear entry of the CUL4B protein, inhibiting the transcription of miR-22-3p and miR-320a, subsequently activating the PI3K/AKT signaling pathway and exerting oncogenic properties in GC (Liu H. T. et al., 2022). Moreover, LINC00152 has shown upregulated expression in GC tissues compared with that in noncancerous tissue samples. Clinical feature analysis demonstrated a significant correlation between LINC00152 expression in GC tissues and pathological features such as tumor size. Functional experiments using shRNA-mediated knockdown of LINC00152 in MGC803 and HGC-27 cells confirmed that its downregulation inhibited cell proliferation. RNA pulldown experiments and RNA immunoprecipitation (RIP) assays showed that LINC00152 interacted with EGF receptor (EGFR) to constitutively activate EGFR and subsequently, the PI3K/AKT signaling pathway, leading to increased tumor cell proliferation (Zhou et al., 2015). Additionally, upregulated expression of LINC01559 in GC tissues was identified to be transmitted from mesenchymal stem cells to GC cells in the form of exosomes. After uptake and entry into GC cells, LINC01559 promotes cell proliferation, migration, and stemness by upregulating phosphoglycerate kinase 1 (PGK1) through miR-1343-3p sponge activity and inducing phosphatase and tensin homolog (PTEN) promoter methylation, resulting in downregulation. These processes ultimately activated the PI3K/AKT pathway, accelerating GC progression (Wang et al., 2020a). Analysis of data from The Cancer Genome Atlas (TCGA) and Gene Expression Omnibus (GEO) online databases revealed that the expression of both LOC101929709 and LIN28B is significantly elevated in GC tissues. LOC101929709, through its interaction with LIN28B, facilitated the recognition and stabilization of m6A-modified c-MYC mRNA, activating the PI3K/AKT pathway and promoting proliferation, migration, and glycolysis in GC cells (Xu et al., 2023). Exosomes derived from *Fusobacterium nucleatum*–infected GC cells (Fn-GCEx) contained increased HOTTIP. Elevated HOTTIP in exosomes acted as an oncogene by sponging miRNA-885-3p, contributing to the overexpression of ephrin type B receptor 2 (EphB2) in uninfected AGS and HGC-27 cells. Further experiments confirmed that EphB2 enhanced GC progression through the activation of the PI3K/AKT pathway (Xin et al., 2023). Moreover, the relative expression of colon cancer–associated transcript-1 (CCAT1) was upregulated in GC tissues. CCAT1 expression exerts promising clinical reference value because of the close correlation with clinicopathological features such as tumor diameter, venous/lymphatic invasion, and clinical biochemical indexes. Cantharidin exerts an antitumor effect by significantly inhibiting the invasion and metastasis of GC cells through the downregulation of CCAT1, thereby blocking PI3K/AKT pathway signaling (Song et al., 2020). Additionally, FOXD1-AS1 exhibits high expression levels in GC cell lines and tissues. FOXD1-AS1 interacts with miR-466, stimulating the PI3K/AKT signaling pathway and increasing FOXD1 protein levels through the eIF4G-eIF4E-eIF4A translation complex. Ultimately, FOXD1 overexpression enhanced cell growth, migration, and chemoresistance of GC cells mediated by FOXD1-AS1 (Wu Q. et al., 2021). Furthermore, upregulated expression of LINC00511 in GC cells promotes cell proliferation, migration, stemness, and the EMT process while inhibiting apoptosis. Mechanistic experiments revealed that the transcription of the oncogene LINC00511 is triggered by SRY-box transcription factor 4 (SOX4). High levels of LINC00511 recruit enhancers of zeste 2 polycomb repressive complex 2 subunit (EZH2) to boost PTEN promoter methylation, inhibiting PTEN expression and driving the PI3K/AKT signaling pathway. Moreover, LINC00511 increases SOX4 expression by sponging miR-195-5p (Wang et al., 2021). However, lncRNA TP53TG1 acted as a tumor suppressor, inhibiting proliferation, metastasis, and cell cycle progression of GC cells while promoting apoptosis. TP53TG1 was downregulated in GC, possibly due to m6A modifications of demethylase ALKBH5 reducing its stability. Low TP53TG1 expression was associated with poor OS, larger tumors, poorer differentiation, advanced TNM stage, and metastasis in patients with GC. TP53TG1 interacts with the oncogenic protein phosphatase 2A inhibitor (CIP2A), triggering its ubiquitination-mediated degradation and inhibiting the PI3K/AKT pathway, underscoring its antitumor significance in GC progression (Fang D. et al., 2022). In summary, these findings suggest that multiple lncRNAs play pivotal roles in regulating various processes of GC cells by modulating the PI3K/AKT signaling pathway.

### 2.2 Liver cancer

Compared with adjacent normal tissues, lncRNA HULC expression is elevated in LC tissues. Experimental findings indicate that HULC plays a role in LC tumorigenesis by boosting the autophagy process, thereby accelerating the proliferation ability of LC cells. Mechanistically, increased HULC levels reduce mature miR15a levels, upregulate autophagy-related protein P62, and enhance the expression of histone deacetylase Sirt1, promoting autophagy in LC cells. Moreover, HULC suppresses PTEN expression through the ubiquitin-proteasome system induced by P62, activating the AKT-PI3K-mTOR signaling pathway (Xin et al., 2018). Strikingly, HULC plays a carcinogenic role together with miR24-2. miR24-2 increases the transcriptional activity of the HULC protein by inhibiting the expression of arginine methyltransferase 7 (PRMT7) in human LC stem cells. The significantly increased HULC further enhances Nanog expression and assists cellular autophagy dependent on the PI3K/AKT pathway (Wang et al., 2020b). Microarray analysis revealed high expression of the lncRNA PTTG3P in HCC tissues compared with that in adjacent normal tissues. Elevated PTTG3P levels in patients with HCC are often associated with unfavorable TNM stage, larger tumors, and poor survival. As for the tumor-promoting mechanism, PTTG3P overexpression upregulates pituitary tumor-transforming 1 (PTTG1), activating the PI3K/AKT pathway and enhancing HCC growth, cell cycle progression, EMT, and metastasis in HCC (Huang J. L. et al., 2018; Li et al., 2022).

**FIGURE 3:**
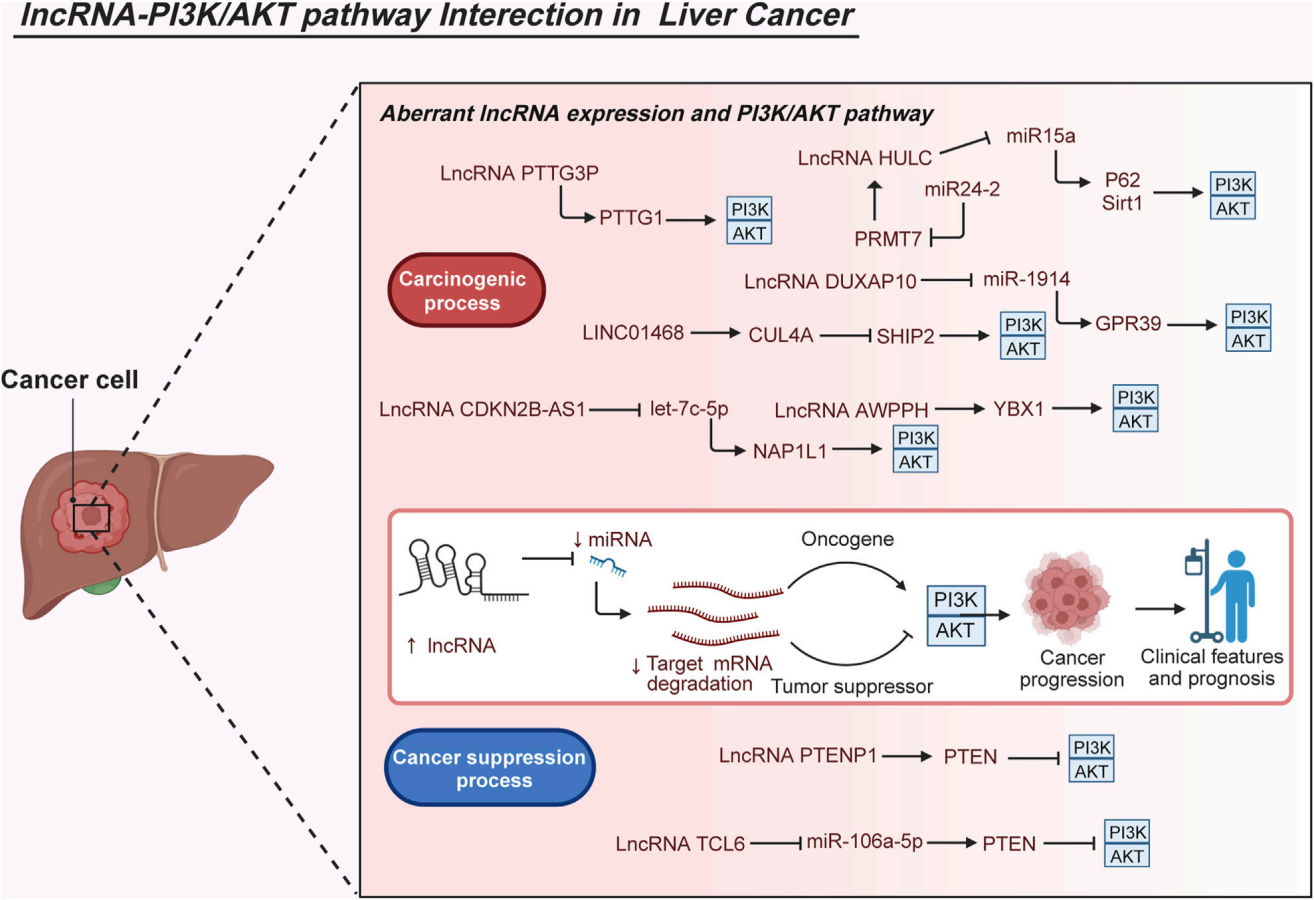
Molecular interactions of abnormal lncRNAs and the PI3K/AKT pathway in liver cancer. The aberrant interplay between lncRNAs and the PI3K/AKT pathway significantly impacts the carcinogenesis of liver cancer. Dysregulated expression of diverse lncRNAs is observed in liver cancer, closely associated with clinical characteristics and patient outcomes. Several studies have elucidated the regulatory mechanisms of lncRNAs in activating the PI3K/AKT pathway during malignant biological processes, crucial for cell growth, metastasis, survival, metabolism, and even drug resistance. Considering their pivotal expression patterns, clinical implications, and roles in carcinogenesis, the interactions between lncRNAs and the PI3K/AKT pathway offer a theoretical foundation for potential future clinical applications such as precise diagnosis, risk stratification, and targeted therapies in liver cancer.

Expression differences of the lncRNA DUXAP10 in HCC and neighboring tissues, showing higher expression in HCC tissues. DUXAP10 overexpression negatively regulates miR-1914, weakening its anticarcinogenic effects. Further experiments involving MHCC-97L and Hep3B cells clarified that DUXAP10 overexpression triggers G-protein–coupled receptor 39 (GPR39) expression and PI3K/AKT signaling, intensifying HCC malignant processes including cell proliferation, colony formation, cell cycle progression, and antiapoptosis (Sun et al., 2019). LncRNA CDKN2B-AS1 is universally overexpressed in several online HCC databases and cell lines, exerting oncogenic functions by enhancing HCC cell proliferation, mobility, migration, and invasion. Moreover, CDKN2B-AS1 has clinical significance owing to its close association with advanced tumor grade, size, stage, vascular invasion, and undesirable OS. CDKN2B-AS1 also acts as a sponge of let-7c-5p to facilitate NAP1L1 expression. Elevated expression of NAP1L1 further stimulates the activation of the PI3K/AKT pathway and promotes the oncogenic role of CDKN2B-AS1 (Huang Y. et al., 2018). Noteworthy, LINC01468 exhibits overexpression in HCC, particularly in cases related to nonalcoholic fatty liver disease–driven HCC. Elevated levels of LINC01468 correspond to poorer outcomes in terms of OS and various unfavorable pathological indicators such as uncontrolled serum biochemical levels, tumor size, TNM stage, and microvascular invasion. Experimental data suggest that enforced expression of PTTG3P significantly accelerates lipid metabolism, subsequently fostering chemoresistance and tumorigenesis. This effect occurs through the facilitation of the Src homology 2-domain-containing PtdIns (3,4,5) P3 5-phosphatase-2 (SHIP2)-cullin 4A (CUL4A) interaction, leading to the ubiquitination degradation of SHIP2. The resultant destabilization of SHIP2 further triggers the activation of the PI3K/AKT/mTOR pathway, thereby contributing to HCC progression (Wang H. et al., 2022). Additionally, the novel lncRNA-AWPPH displays heightened levels in HCC tissues and is deemed an independent prognostic factor in HCC. Its elevation is positively associated with disease-free survival, recurrence-free survival, and OS rates among patients with HCC. The lncRNA-AWPPH interacts with YBX1, facilitating the transcriptional activation of the PI3K/AKT pathway. These findings underscore the malignant roles of lncRNA-AWPPH, including its involvement in promoting cell proliferation and migration, tumor growth, and metastasis (Zhao et al., 2017). Notably, decreased levels of lncRNA PTENP1 were observed in highly metastatic HCC Mahlavu cells through quantitative reverse transcription–polymerase chain reaction (qRT-PCR) analysis. Ectopic PTENP1 expression through Sleeping Beauty–based hybrid baculovirus vectors significantly augments PTEN levels, dampens the activation of the PI3K/AKT pathway, induces autophagy and apoptosis, and suppresses cell proliferation, migration, invasion, angiogenesis, and neo vasculature maturation (Chen et al., 2015). Furthermore, the lncRNA TCL6 displays conspicuous downregulation in HCC tissues derived from the GEO dataset (GSE14520) and in HepG2, SMMC-7721, and MHCC-97H cells. Functioning as a tumor-suppressive lncRNA, TCL6 curbs the carcinogenic effects of the PI3K/AKT signaling pathway by directly interacting with miR-106a-5p, subsequently upregulating PTEN expression in HCC (Luo et al., 2020). In cholangiocarcinoma, studies demonstrate abnormal enrichment of lncRNA HCG18 in tumor tissues sourced from the TCGA database and QBC939 cells. Functional experiments affirm the cancer-promoting role of HCG18, influencing cell viability, migration, and invasion as well as the EMT process in QBC939 cells. Additionally, investigations indicate the overexpression of HCG18 in exosomes released by these cells, leading to reduced expression of miR-424-5p in surrounding cancer cells. Decreased miR-424-5p levels subsequently elevate the expression of SRY-box transcription factor 9 (SOX9) and activate the PI3K/AKT pathway, culminating in accelerated tumor progression in cholangiocarcinoma (Ni et al., 2023). Additionally, in hepatoblastoma tissues, analysis based on the GEO database showed upregulated expression of the lncRNA MIR205HG. This upregulation was confirmed in the cell cytoplasm of HepG2 and HuH-6 cells. Functional assays demonstrated that decreased MIR205HG expression hindered the proliferation, migratory, and invasive potential of HepG2 and HuH-6 cells. Furthermore, a series of experiments indicated that MIR205HG acts as a strong sponge for miR-205-5p, thereby enhancing the activity of MAPK and the PI3K/AKT pathway (Zhang et al., 2022). These observations collectively illustrate how lncRNAs intricately activate the PI3K/AKT pathway through complex mechanisms, thereby modulating various pathological functions such as apoptosis, proliferation, migration, and invasion in LC cells (Figure 3).

### 2.3 Colorectal cancer

LINC01296 exhibits a remarkable increase in CRC and shows a close association with advanced tumor stage and distant metastasis. Kaplan-Meier analysis further illustrates that elevated levels of LINC01296 correlate with poor clinical outcomes in patients with CRC. LINC01296 levels significantly influence the proliferation and invasion abilities of CRC cell lines and affect their resistance to 5-fluorouracil (5-FU) chemotherapy. Mechanistically, LINC01296 directly diminishes the levels of miR-26a, thereby augmenting the expression of GALNT3, a key enzyme involved in O-glycosylation. This elevation of GALNT3 subsequently enhances the O-glycosylation of mucin1 (MUC1) and activates the PI3K/AKT signaling pathway. The crosstalk of LINC01296-miR-26a-GALNT3-MUC1-PI3K/AKT underscores its role in promoting tumor growth, liver metastasis, and conferring chemo-resistance to 5-FU in CRC (Liu et al., 2018). Additionally, high levels of lncRNA HOTAIR are observed in CRC tissues and cell lines. HOTAIR appears to sponge miR-326, leading to increased levels of fucosyltransferase 6 (FUT6) and subsequent facilitation of CD44 fucosylation. The α1, 3-fucosylated CD44, in turn, amplifies the PI3K/AKT pathway, mediating HOTAIR’s pro-tumorigenic effects in CRC, such as accelerated cell proliferation, migration, invasion, tumorigenesis, and distant metastasis. Kaplan-Meier analysis also confirms the association between high HOTAIR expression and poor prognosis in CRC (Pan et al., 2019). Additionally, elevated levels of lncRNA TCONS_00012883 are observed in CRC tissues, as confirmed by qRT-PCR analysis. This study demonstrates that TCONS_00012883 exhibits higher expression in tumor samples with advanced tumor stages and metastasis. Moreover, Kaplan-Meier survival analysis reveals that patients with elevated levels of TCONS_00012883 experience shorter OS and progression-free survival. *In vitro* and *in vivo* experiments further establish that TCONS_00012883 amplifies cell proliferation and metastasis, thus enhancing tumor growth and metastasis. TCONS_00012883, primarily localized in the cell nucleus, upregulates the target gene matrix metallopeptidase 1 (MMP1) by interacting with DEAD-box helicase 3 (DDX3) protein and mediating reverse activation of the transcription factor YY1. This interaction ultimately contributes to the activation of the PI3K/AKT pathway, mediating the tumor-promoting functions of TCONS_00012883 in CRC (Yang et al., 2020). Recent studies have highlighted the significant increase in LINC00659 levels within CRC tissues, linking it to an oncogenic role in lncRNA. This upsurge in LINC00659 expression is associated with shortened patient survival. Knockdown of LINC00659 disrupts the regulation of the PI3K-Akt signaling pathway, exerting a suppressive influence on CRC cell cycle progression, proliferation, drug resistance, and colony formation (Tsai et al., 2018). Furthermore, lncRNA microarray analysis revealed elevated small nucleolar RNA host gene 7 (SNHG7) expression profiles in CRC tissues and metastatic CRC cell lines. SNHG7 promotes malignant progression both *in vitro* and *in vivo*. High SNHG7 levels correlate with adverse tumor characteristics such as size, lymphatic metastasis, distant metastasis, tumor stage, and OS. SNHG7 interacts with miR-34a, reducing its expression through a ceRNA mechanism. Consequently, the downstream acetylgalactosaminyltransferase GALNT7 is upregulated, causing abnormal O-glycosylation and activating mTOR, thereby initiating the PI3K/Akt pathway (Li et al., 2018). Moreover, highly metastatic CRC cells release exosomes containing elevated levels of MALAT1. These exosome-derived MALAT1 molecules enhance the proliferative, invasive, and metastatic capabilities and confer resistance to 5-FU in primary CRC cells. MALAT1 interacts with miR-26a/26b, elevating fucosyltransferase 4 (FUT4) levels and related fucosylation. This interaction further activates the PI3K/AKT/mTOR pathway, thereby mediating CRC progression and metastasis (Xu et al., 2020). The lncRNA AB073614 has shown differential expression in CRC cells and tissues from patients with CRC. Elevated AB073614 levels in CRC tissues, when compared with paired normal tissues, are closely associated with adverse clinical pathological features such as tumor grade, size, cell differentiation status, and distant metastasis. Knockdown of AB073614 in SW480 cells significantly impedes the PI3K/AKT pathway, inhibiting proliferation, migration, and invasion while promoting apoptosis and G1-phase cell cycle arrest (Wang Y. et al., 2017). The lncRNA ST3GAL6-AS1 has demonstrated inhibitory effects on CRC progression through glycosylation processes. Its expression level shows a negative correlation with key aspects such as tumor size, lymph node metastasis, distant metastasis, and tumor stage. In CRC cells, ST3GAL6-AS1 triggers the transcription of ST3GAL6, leading to an elevation in α-2,3 sialylation levels of glycoproteins. This, in turn, inhibits the activation of the PI3K/Akt signaling pathway and phosphorylation of Forkhead box1 (FOXO1). These outcomes underscore the significance of interactions involving ST3GAL6-AS1, ST3GAL6, PI3K/Akt, and FOXO1 in regulating CRC progression. Understanding these mechanisms holds promise for developing therapeutic strategies against CRC (Hu et al., 2019). Additionally, analysis of CRC specimens in the TCGA and GTEx databases revealed decreased expression of LINC02038 in CRC tissues compared with that in adjacent para-cancerous tissues. Low LINC02038 expression correlates positively with higher invasiveness, distant metastasis, higher TNM staging, and poorer OS of patients. In LoVo and SW620 cells, LINC02038 functions as a molecular sponge for miR-552-5p, thereby inhibiting the degradation of FAM172A. This inhibition suppresses the activation of the PI3K/AKT pathway. These findings strongly indicate the inhibitory roles of LINC02038 in CRC cell proliferation, vitality, migration, and invasive abilities (Liu W. et al., 2023).

### 2.4 Pancreatic cancer

Research has highlighted the significant role of LINC01268 contained in exosomes, emphasizing its pivotal involvement in tumor growth and invasion within the context of PC. Specifically, LINC01268 operates as an oncogene, orchestrating malignant processes such as cell proliferation, migration, EMT, and invasion in PC. RNA-seq analysis uncovered a substantial upsurge in LINC01268 expression in plasma samples obtained from patients with PC, a finding further corroborated through qRT-PCR analysis conducted on PC tumor tissues. Subsequent investigations unveiled that elevated LINC01268 expression leads to a reduction in miR-217 levels and an increase in KIF2A expression in PC cells AsPC-1 and PANC-1. These alterations trigger the activation of the PI3K/AKT pathway, consequently fueling tumor cell growth, proliferation, migration, invasion, and EMT progression (Liu S. et al., 2023). Moreover, analysis from the TCGA database indicates a notably high expression of the lncRNA HOXA10-AS in PC tissues. Survival analysis underscores the substantial prognostic value of HOXA10-AS for patients with PC. Further exploration has illuminated the indispensable role of HOXA10-AS in driving the malignant biological behavior of PC, largely mediated by the PI3K-AKT pathway. Molecular analysis confirms that HOXA10-AS acts as a sponge for miR-340-3p, thereby augmenting the expression of the hydroxytryptamine receptor 1D (HTR1D). This regulatory mechanism significantly affects the proliferation, migration, and differentiation of PC cells by upregulating the PI3K-AKT pathway (Wu et al., 2022). Similarly, LINC01094 exhibits significant overexpression in PC tissues based on the GEO database and correlates with unfavorable clinical features, including tumor size, lymphatic metastasis, and TNM classification. Silencing of LINC01094 in xenograft mouse models notably reduces the proliferation, migration, and metastasis of PC cells, elucidating its role in promoting tumor growth and metastasis. Functional experiments of the carcinogenic mechanism have revealed that LINC01094 serves as an endogenous sponge for miR-577, elevating the RNA-binding protein lin-28 homolog B (LIN28B) levels, thereby activating the PI3K/AKT pathway (Luo C. et al., 2021). Additionally, marked downregulation of lncRNA MEG3 has been observed in tissue samples from patients with PC. Reduced MEG3 expression aligns with more aggressive tumor characteristics such as increased size, metastasis, and vascular invasion. Cellular experiments have confirmed the suppressive role of MEG3 in PC by inhibiting the PI3K/AKT signaling pathway, resulting in impaired cell proliferation, invasive capacity, and migration (Gu et al., 2017). Furthermore, microarray analysis of six human PC tissues has identified aberrantly decreased LINC00671 expression. Patients exhibiting lower LINC00671 levels typically present adverse tumor differentiation and TNM staging, correlating with poor outcomes. Both *in vitro* and *in vivo* experiments have demonstrated the involvement of LINC00671 in inhibiting the PI3K-Akt signaling pathway, thereby restraining PC cell proliferation, invasion, and migration (Qu et al., 2021). These collective findings underscore the pivotal role of lncRNAs in regulating PC progression by modulating the activity of the PI3K pathway. This discovery provides a promising avenue for potential targeted therapies in the treatment of PC.

### 2.5 Esophageal cancer

Whole-genome sequencing and RNA sequencing analysis of EC have identified significant mutation patterns in multiple lncRNAs and frequently abnormal PI3K/AKT pathways. These findings suggest their pivotal roles in the occurrence and development of EC (Chang et al., 2017). Further functional assays are needed to validate the interaction between lncRNAs and the PI3K/AKT pathway for precision treatment and prevention of EC in patients. Multiple studies indicate that dysregulated lncRNAs contribute to chemotherapy resistance across various types of cancer. Recent research specifically highlights LINC01014’s involvement in regulating drug resistance in EC. Overexpression of LINC01014 notably inhibits gefitinib-induced apoptosis in FLO-1 cells. Additionally, silencing of LINC01014 in gefitinib-resistant FLO-1 cell lines significantly enhances the chemosensitivity of gefitinib-resistant FLO-1 cells to gefitinib, impairing the PI3K-AKT-mTOR signaling pathway (Fu et al., 2020). Moreover, the lncRNA MCEI (lncRNA mediated the chemosensitivity of ESCC by regulating IGF2) plays a role in the tumorigenesis of EC and suppresses EC cell chemosensitivity to cisplatin. Analysis of the GSE89102 dataset identifies MCEI as significantly upregulated in EC tissues. Further assessment using the TCGA dataset reveals a strong association between MCEI expression and reduced OS rates in patients with EC. MCEI binds to miR-6759-5p, increasing IGF2 concentration, thus promoting AKT phosphorylation. These findings highlight the oncogenic functions of MCEI, mediating EC cell chemosensitivity to cisplatin through the miR-6759-5p/IGF2/PI3K/AKT axis (Liu G. et al., 2020). Furthermore, high expression of lncRNA CASC9 correlates with worse tumor differentiation, greater depth of primary tumor infiltration, lymph node metastasis, and advanced TNM staging in EC. Prognostic analysis confirms the association between high CASC9 expression and poor OS in patients with EC. Additional analysis reveals that CASC8 enhances CREB-binding protein (CBP) enrichment in the LAMC2 promoter region, upregulating LAMC2 expression in EC KYSE150 and KYSE450 cells. Subsequently, upregulated expression of LAMC2 activates focal adhesion kinase, PI3K, and AKT phosphorylation, facilitating the pro-metastatic effects mediated by CASC9 in EC progression (Liang et al., 2018). Moreover, dysregulation of the lncRNA SNHG17 closely associates with the EMT process in EC development. Upregulated expression of SNHG17 induced by TGF-β1 in EC Eca109 cells directly regulates c-Myc transcription, activating the PI3K/AKT signaling pathway and promoting cell proliferation, migration, invasion, and EMT. High SNHG17 expression correlates positively with poorer survival and unfavorable clinicopathologic features of patients with EC in the GEO dataset (GSE20347) and GEPIA (Shen et al., 2022). Additionally, high expression of lncRNA ESCCAL-1 in EC tumors is associated with poorer OS, disease-free survival (DEF), and clinicopathological indexes. Knockdown of ESCCAL-1 in EC-9706 and EC-109 cells inhibits malignant cellular processes, including proliferation, migration, invasion, and resistance to apoptosis. Mechanistic experiments suggest that ESCCAL-1 acts as a sponge for miR-590-3p, preventing the suppression of APOBEC3G expression by miR-590-3p. This action activates the PI3K/Akt signaling pathway, thereby promoting malignant characteristics induced by ESCCAL-1 in EC (Liu J. et al., 2020). These results underscore the critical role of the interaction between lncRNAs and miRNAs, along with their involvement in the PI3K/AKT pathway, in the progression of EC.

## 3 Clinical significance of lncRNAs and the PI3/AKT pathway in gastrointestinal tumors

Recent studies have unveiled the dysregulation of diverse lncRNAs in gastrointestinal tumors, closely linked with clinical and pathological features as well as patient prognosis (Huang et al., 2023; Ma and Hu, 2023; Samadi et al., 2023; Xiao et al., 2023). Experimental findings underscore the pivotal regulatory roles of lncRNAs in the initiation and progression of gastrointestinal tumors, influencing vital biological behaviors such as cell proliferation, migration, invasion, and chemoresistance (Khajehdehi et al., 2021). Notably, lncRNAs often act as ceRNAs, binding with miRNAs to modulate mRNA expression of downstream target genes, subsequently affecting the PI3K/AKT pathway’s activity (Liu et al., 2017; Zhang et al., 2017; Lee et al., 2019; Qian et al., 2019; Ghafouri-Fard et al., 2021). In CRC, the ceRNA network formed by lncRNA/miRNA/mRNA interaction plays a vital role in biological processes such as EMT, metastasis, and treatment resistance (Wang et al., 2019a). The altered activity of the PI3K/AKT pathway, mediated by lncRNAs, plays a crucial role in tumor development (Duan et al., 2018; Luo et al., 2019; Duan et al., 2020; Fattahi et al., 2020; Shang et al., 2023). The intricate interplay between lncRNAs and the PI3K/AKT pathway offers crucial insights into gastrointestinal tumor progression (Moafian et al., 2021). Molecular targeted therapy has made substantial strides in cancer treatment, emphasizing the increasing importance of targeting lncRNAs or the PI3K/AKT signaling pathway for therapeutic intervention for gastrointestinal tumors (Gao et al., 2022; Mortazavi et al., 2022; Maharati and Moghbeli, 2023). However, developing drugs targeting specific lncRNAs is challenging because of their diverse structures and functions, complicating a comprehensive understanding of their precise mechanisms (Ferrè et al., 2016; Kopp and Mendell, 2018; Ali and Grote, 2020; Bridges et al., 2021; Herman et al., 2022). Additionally, the dual capacity of lncRNAs to promote and inhibit tumor growth adds complexity to their clinical application in gastrointestinal cancer therapy. Moreover, evidence suggests limited single-agent efficacy of inhibitors targeting PI3K or AKT under tolerable doses (Liu et al., 2009; Cho, 2014; Fruman and Rommel, 2014). Moreover, it remains uncertain whether available PI3K or AKT inhibitors effectively inhibit the pathway for anti-tumor effects. The lack of a qualified biomarker hampers identifying responsive patient populations and projecting clinical benefits from PI3K inhibitors (Rodon et al., 2013; Polivka and Janku, 2014; Yang et al., 2019). The association between lncRNA and PI3K signaling pathways involves multiple cellular signaling pathways. By intervening in specific lncRNA-PI3K signaling pathway interactions, it is possible to simultaneously target multiple downstream signaling pathways, which increases the difficulty of targeted drug development. Further comprehensive experimental and clinical studies are essential to grasp the molecular mechanisms and clinical significance of lncRNA-PI3K/AKT pathway interactions fully.

Identifying specific patterns of lncRNA-PI3K/AKT pathway interactions in gastrointestinal tumors not only lays a theoretical foundation for related therapeutic avenues but also aids in discovering valuable biomarkers for disease progression and prognosis. Multiple studies highlight significantly elevated levels of various lncRNAs in the plasma or tissues of patients with gastrointestinal tumors (Bhan et al., 2017). The detection of specific lncRNA levels in the plasma or tissues can serve as novel sensitive biomarkers for diagnosis. For instance, three lncRNAs (NEAT1:11, lnc-PDZD8-1:5, and LINC00910:16) exhibited significant upregulation in CRC through RNA sequencing. Combined detection of these lncRNAs in peripheral blood mononuclear cells demonstrated 74.5% sensitivity and 84.5% specificity in diagnosing CRC (Li et al., 2023). Online databases such as TCGA and GEO offer rich patient resources, facilitating the identification of prognosis-related lncRNAs that can evaluate adverse clinical characteristics and prognoses, aiding in personalized treatment strategies (Charles and Eichhorn, 2018; Wu Z. et al., 2021; Grammatikakis and Lal, 2022). Univariate and multivariate analyses have revealed the independent prognostic potential of multiple lncRNAs in predicting digestive tract cancer prognosis. For instance, lncRNA AWPPH expression levels serve as an independent prognostic factor for assessing recurrence-free and OS rates in patients with HCC (Zhao et al., 2017). Similarly, the lncRNA PTTG3P predicts the prognosis of patients with HCC independently. The upregulated expression of SNHG17 serves as an independent prognostic marker (Shen et al., 2022). In GC, expression levels of the lncRNA AK023391 independently predict the clinical outcomes of patients with GC, while the lncRNA TP53TG1 acts as a potential indicator for early diagnosis and prognosis (Huang et al., 2017; Fang D. et al., 2022). Additionally, lncRNA THAP7-AS1 expression levels can differentiate whether patients with GC experience lymph node metastasis (Liu H. T. et al., 2022). These collective studies demonstrate the significant clinical diagnostic and prognostic value of lncRNAs in gastrointestinal tumors. In light of the positive outcomes from current research, the potential therapeutic impact of interactions between lncRNA and PI3K as well as their role as diagnostic and prognostic indicators for gastrointestinal tumors is evident.

Given the non-selectivity of the PI3K-AKT pathway, several innovative strategies can be expected to improve the treatment efficacy of PI3K-AKT pathway inhibitor (Asati et al., 2016; Fattahi et al., 2020; Mortazavi et al., 2022; Khezri et al., 2024). One promising approach involves developing inhibitors that specifically target mutations or alterations in the PI3K-AKT pathway unique to tumor cells (Adil et al., 2021; Leiphrakpam and Are, 2024; Morgos et al., 2024). Utilizing gene-editing technologies such as CRISPR/Cas9 to selectively knock out components of the PI3K-AKT pathway in tumor cells while sparing normal cells offers high specificity for precise targeting of cancer cells without affecting healthy tissue (Matano et al., 2015; Faulkner et al., 2020; Bao et al., 2022; Singh et al., 2023). Similarly, nanoparticle-based delivery systems can be designed to selectively deliver PI3K-AKT pathway inhibitors to tumor cells (Sandhiutami et al., 2020; Du A. et al., 2022; Wang R. et al., 2022; Sanaei et al., 2022). Another effective strategy is combining PI3K-AKT pathway inhibitors with other treatments that are more selective for tumor cells, such as targeted therapies against tumor-specific antigens or immunotherapies (Fresno et al., 2004; Alzahrani, 2019; Fattahi et al., 2020; Yu et al., 2022). This combined approach can improve the overall specificity and efficacy of the treatment by attacking the cancer cells through multiple mechanisms. Crucial to these strategies is the identification and validation of biomarkers that predict responsiveness to PI3K-AKT pathway inhibitors (Huang T. et al., 2018; Xu et al., 2024). By stratifying patients based on the presence of these biomarkers, treatments can be personalized and applied more selectively, ensuring that patients most likely to benefit from PI3K-AKT inhibitors receive them.

Extensive studies have been conducted on various inhibitors and clinical drugs targeting the PI3K-AKT pathway as RNA based cancer gene therapy, including their applications in gastrointestinal tumors (Table 3). Both basic and clinical trials have evaluated the efficacy of these inhibitors, including PI3K inhibitor Alpelisib, AKT inhibitor Ipatasertib, and dual PI3K/mTOR inhibitor Dactolisib (Saura et al., 2017; Bang et al., 2019; Yu et al., 2020; Xu et al., 2021; Liao et al., 2023). These inhibitors represent promising treatment options for gastrointestinal tumors, either as monotherapies or in combination with other therapies. Currently, there are some clinical trials have investigated inhibitors targeting this pathway to determine their efficacy and safety in different types of cancers. For example, a Phase II trial explores the PI3K inhibitor SF1126 in treating patients with advanced or metastatic cancer (ClinicalTrials.gov IDNCT02644122).

**TABLE 3:** Key clinical trials on inhibiting of PI3K-Akt signaling pathways and RNA based cancer gene therapy.

ClinicalTrials.gov ID	Official Title	Phase	Intervention/Treatment	Target	Study design	Conditions	Enrollment
NCT01816984	A Biomarker Driven Pilot Study of the Pan-class I PI3K Inhibitor NVP-BKM120 in Combination With Cetuximab in Patients With Recurrent/Metastatic Head and Neck Cancer	Phase I/II	BKM120	PI3K	Combination therapy with Cetuximab	Recurrent or Metastatic Head and Neck Cancer	12
NCT02874404	A Single-Center Phase IIa Study Evaluating the Safety and Tolerability of TGR-1202 and Ibrutinib in Patients With Relapsed or Refractory Diffuse Large B-Cell Lymphoma: A Trial of the Lymphoma Precision Medicine Laboratory	Phase II	TGR-1202	PI3K	Combination therapy with Ibrutinib	Relapsed or Refractory Diffuse Large B-Cell Lymphoma	13
NCT01390818	An Open-Label, Phase Ib Dose Escalation Trial of Oral Combination Therapy With MSC1936369B and SAR245409 in Subjects With Locally Advanced or Metastatic Solid Tumors	Phase I	SAR245409	PI3K	Combination therapy with Pimasertib	Locally Advanced or Metastatic Solid Tumors	146
NCT05676710	A Single-arm, Open-label, Pilot Study on the Efficacy and Safety of PI3K Inhibitors in Relapsed/Refractory Large Granular T Lymphocytic Leukemia	Phase I	Linperlisib	PI3K	Monotherapy	Relapsed/Refractory Large Granular T Lymphocytic Leukemia	8
NCT01723800	Phase I Trial of BKM120 in Combination With Carboplatin and Pemetrexed in Patients With Advanced Non-Squamous Non-Small Cell Lung Cancer (NSCLC)	Phase I	BKM120	PI3K	Combination therapy with Carboplatin and Pemetrexed	Stage IV Non-Small Cell Lung Cancer	9
NCT01882803	A Phase 2 Study of Duvelisib in Subjects With Refractory Indolent Non-Hodgkin Lymphoma	Phase II	Duvelisib	PI3K	Monotherapy	Refractory Indolent Non-Hodgkin Lymphoma	129
NCT01613950	A Phase IB, Multicenter, Open-label Dose Escalation Study of the PI3K Inhibitor BYL719 in Combination With the HSP90 Inhibitor AUY922 in Patients With Advanced or Metastatic Gastric Cancer Carrying a Molecular Alteration of PIK3CA or an Amplification of HER2	Phase I	BYL719	PI3K	Combination therapy with AUY922	Advanced or Metastatic Gastric Cancer	18
NCT01540253	A Phase I Study of the PI3-Kinase Inhibitor BKM120 in Combination With Docetaxel in Patients With Advanced Solid Tumors	Phase I	BKM120	PI3K	Combination therapy with Docetaxel	Advanced Solid Tumor That is Locally Advanced, Cannot Be Removed By Surgery, or Metastatic	38
NCT02929797	An Open Label Continuation Study of the Oral AKT Inhibitor GSK2110183 in Subjects With Solid Tumors and Hematologic Malignancies	Phase II	GSK2110183	AKT	Monotherapy	Solid Tumors and Hematologic Malignancies	11
NCT00920257	A Phase I, Open-Label, Two-Stage Study to Investigate the Safety, Tolerability, Pharmacokinetics and Pharmacodynamics of the Oral AKT Inhibitor GSK2141795 in Subjects With Solid Tumors or Lymphomas	Phase I	GSK2141795	AKT	Monotherapy	Solid Tumors or Lymphomas	77
NCT01266954	An Open Label Study To Investigate the Pharmacokinetics and Pharmacodynamics of Repeat Escalating Doses of the Oral AKT Inhibitor GSK2141795 by 18F FDG PET Analysis in Subjects With Ovarian Cancer	Phase I	GSK2141795	AKT	Monotherapy	Ovarian Cancer	36
NCT01476137	An Open-Label, Two Part, Phase I/II Study to Investigate the Safety, Pharmacokinetics, Pharmacodynamics, and Clinical Activity of the MEK Inhibitor GSK1120212 in Combination With the AKT Inhibitor GSK2110183 in Subjects With Solid Tumors and Multiple Myeloma	Phase I/II	GSK2110183	AKT	Combination therapy with GSK1120212	Solid Tumors and Multiple Myeloma	335
NCT01333475	Pilot Study of the Combination of MK-2206, an AKT Inhibitor, and AZD6244, a MEK Inhibitor, in Patients With Advanced Colorectal Carcinoma	Phase II	MK-2206	AKT	Combination therapy with AZD6244	Advanced Colorectal Carcinoma	21
NCT01231919	A Phase I Study of MK-2206, an AKT Inhibitor, in Pediatric Patients With Recurrent or Refractory Solid Tumors or Leukemia	Phase I	MK2206	AKT	Monotherapy	Recurrent or Refractory Solid Tumors or Leukemia	45
NCT02017509	A Study to Determine the Immunopheotype of Locally Advanced Rectal Adenocarcinoma and Its Correlation With the Efficacy of Neoadjuvant Chemoradiotherapy	Observational	RNA gene expression analysis	RNA-based	—	Rectal Cancer	3
NCT01787994	A Phase I, Open Label, Dual Cohort, Single Center Study to Evaluate the Safety, Tolerability and Immunogenicity of Autologous CD4 T Cells Modified With a Retroviral Vector Expressing the MazF Endoribonuclease Gene in Patients With HIV	Phase I	Autologous CD4^+^ T cells genetically modified with a retroviral vector expressing the MazF endoribonuclease gene (MazF-T)	RNA-based	—	HIV	10
NCT02455089	Prognosis Assessment of the Increase of GADD34 Gene Expression for Patient Suffering From Systemic Lupus Erythematosus	—	GADD34 RNA level measurement	RNA-based	—	Systemic Lupus Erythematosus	143
NCT03282656	Pilot and Feasibility Study of Hematopoietic Stem Cell Gene Transfer for Sickle Cell Disease	Phase I	single infusion of autologous bone marrow derived CD34^+^ HSC cells transduced with the lentiviral vector containing a short-hairpin RNA targeting BCL11a	RNA-based	—	Sickle Cell Disease	10
NCT03197805	Prospective Study Assessing the Impact of RNA Genomic Profile Defined by a Genomic Test on Treatment Decision-making in Breast Cancer Patients With an ISH Equivocal HER2 Status- EQUIVOK Study	—	PAM 50 test	RNA-based	—	HER2 Equivocal Breast Cancer	26
NCT05373251	PATH Trial: Personalized Approaches in the Treatment of Head and Neck Cancer	—	Whole genomic DNA/RNA tumour sequencing	RNA-based	—	Head and Neck Cancer	500
NCT04476537	Identification of Personalized Treatment Utilizing Master Regulator Gene Targets in Pancreatic Cancer	—	OncoTreat	RNA-based	—	Advanced Pancreatic Cancer	30
NCT03480152	A Phase I/II Trial to Evaluate the Safety and Immunogenicity of a Messenger RNA (mRNA)-Based, Personalized Cancer Vaccine Against Neoantigens Expressed by the Autologous Cancer	Phase I/II	National Cancer Institute (NCI)-4650, a messenger ribonucleic acid (mRNA)-based, Personalized Cancer Vaccine	RNA-based	—	Autologous Cancer	5
NCT00834002	Wilms Tumor Gene (WT1) mRNA-transfected Autologous Dendritic Cell Vaccination for Patients With Acute Myeloid Leukemia (AML): a Phase I Trial	Phase I	injection of cultured dendritic cells loaded with mRNA coding for WT1				10

Despite emerging research on the mechanisms between lncRNAs and the PI3K-AKT pathway, there remains a lack of effective inhibitors targeting the interaction between lncRNAs and the PI3K-AKT pathway. Given the vital role of the interaction between lncRNAs and the PI3K-AKT pathway in gastrointestinal tumors, further research is essential to fill this gap and develop viable treatment strategies that can significantly improve patient outcomes.

## 4 Conclusion

Gastrointestinal tumors remain a formidable challenge within public health. Numerous studies have demonstrated the critical role of the interplay between lncRNAs and the PI3K/AKT pathway to the carcinogenesis of gastrointestinal tumors. The dysregulated expression of lncRNAs, primarily through sponging specific miRNAs to regulate downstream target genes, significantly influences the activity of the PI3K/AKT pathway. This involvement plays a crucial role in the initiation and progression of gastrointestinal tumors, resulting in uncontrolled cell proliferation, invasion, reduced apoptosis, metastasis, and angiogenesis. Moreover, prior research has underscored the correlation between aberrant lncRNA expression levels and the clinical characteristics and outcomes of patients with gastrointestinal tumors, suggesting that lncRNAs have significant potential as vital diagnostic and prognostic markers.

In recent years, the burgeoning exploration of lncRNAs in gastrointestinal tumors has underscored its significance as a plausible therapeutic target. As previously reported, targeting lncRNAs and the PI3K/AKT pathway offers a promising avenue to counter chemoresistance and enhance the effectiveness of tumor treatment. This interaction presents an innovative therapeutic approach deserving of further investigation within the context of gastrointestinal tumors. Through a deeper exploration of the underlying mechanisms of lncRNAs and the PI3K/AKT pathway in these tumors, coupled with the development of targeted therapeutic interventions, we can envision substantial advancements in precise diagnosis, prognosis, and treatment methodologies for gastrointestinal tumors.
